# Chilean Market Protein Shakes Composition

**DOI:** 10.3390/nu16081129

**Published:** 2024-04-11

**Authors:** Carlos Jorquera, Guillermo Droppelmann, Paula Pridal, Javier Faúndez, Felipe Feijoo

**Affiliations:** 1 Facultad de Ciencias, Escuela de Nutrición y Dietética, Universidad Mayor, Santiago 8580745, Chile; carlos.jorquera@mayor.cl (C.J.); paula.pridal@meds.cl (P.P.); 2 Clínica MEDS, Santiago 7550000, Chile; 3 Harvard T.H. Chan School of Public Health, Boston, MA 02115, USA; 4 Club Social y Deportivo Unión Española, Santiago, Chile; javierfaundeza@gmail.com; 5 School of Industrial Engineering, Pontificia Universidad Católica de Valparaíso, Valparaíso 2340025, Chile; felipe.feijoo@pucv.cl

**Keywords:** amino acids, BCAA, ergogenic, market, sport diet, whey protein

## Abstract

Understanding the nutritional content of protein supplements is crucial for optimal nutritional planning among athletes and other people. Distribution of macronutrients and aminograms in the main products available in the national Chilean market remains unknown. A descriptive cross-sectional study was conducted to identify the main protein supplements available in the Chilean market. Information on macronutrients and aminograms from the nutritional labels of each product was extracted. The analysis considered the content per portion and per 100 g. Cluster analysis models and graphical representations were explored. Eighty protein shakes were assessed in the Santiago de Chile market. The median protein dosage was 32 g (range from 25 to 52), and the median energy value stood at 390 kcal (range from 312 to 514). The median protein content per 100 g of product was found to be 75 g (range from 42.5 to 97.2). The combined median concentration of amino acids was 4749.75 mg. Among these, the essential amino acid L-Tryptophan exhibited the lowest concentration at 1591.50 mg, while the conditional amino acid L-Glutamine had the highest median concentration at 17,336 mg. There was a significant prevalence of animal-derived products, placing specific emphasis on protein supplements that feature elevated levels of the amino acids L-Glutamine and L-Leucine.

## 1. Introduction

Physical inactivity, sedentary behaviors, and obesity are considered some of the most critical public health problems of the 21st century [1]. Indeed, after the COVID-19 pandemic, people’s physical activity and quality of life decreased significantly [2]. Nonetheless, there is an increasing interest among the population in consuming products aimed at enhancing physical well-being and at supporting rapid recovery [3]. In the years 1999–2012, it was estimated that, in the United States, more than half of adults consumed some nutritional supplement in the last 30 days [4]. Recently, the Council for Responsible Nutrition released the results of its annual survey on the use of dietary supplements [5]. The survey found that more than 75% of consumers in North America took some nutritional supplement and that sports nutrition supplements have increased by 5 points since last year, to 39% [5]. In particular, whey protein supplements (WPS) have grown in popularity over the past ten years and are the best-selling protein powder. Additionally, the global protein supplements market is expected to expand by 8% from 2022 to 2030 [6].

Despite the fact that, like other countries, such as the United States, Chile does not have specific regulations for the nutritional report of the WPS, the country has led a series of initiatives to improve the information on nutritional components. For example, in 2012, Law 20,606 was promulgated, representing the first global mandatory labeling policy to report the nutritional components of all foods sold in the country. The purpose was to develop a phased public health strategy to increase the requirements and reduce the critical nutrients of foods whose energy, sugar, salt, and fat content were outside the nutrient limits, according to the recommendations of the Chilean Ministry of Health [7].

Recently, multiple health benefits of the Chilean nutritional warning label initiative have been reported in the international literature [8,9]. However, the nutritional information available in the Chilean market concerning WPS is limited. In 1997, the first national cycle of regulation of these products began with the publication of Title XXIX of the Food Sanitary Regulations, with a recent update in 2019. The document describes the conceptual orientation of the labels and the maximum doses allowed for human consumption [10]. At the same time, despite the fact that Law 20,606 presented an update in August 2021, the efforts of local authorities are still insufficient to regulate nutritional information regarding WPS. 

On the other hand, the impact of protein consumption on a healthy person is critical for achieving functional levels of behavior in various systems. Proteins are composed of amino acids (AA) linked by peptide bonds, which, when hydrolyzed, are absorbed by the intestine, allowing entry into the musculoskeletal system and other tissues necessary for the structural and functional systems of the human body. The amount of these and/or constituent amino acids that the diet must provide to meet metabolic demand determines the dietary requirement for proteins [11]. In this regard, there are various recommendations for individuals’ daily protein intake, with the definition of the exact moment when muscles begin to age due to a gradual decrease in muscle mass being controversial [12]. However, authors have indicated that protein intake is important throughout the life cycle, making specific estimates according to age range, determining the amount of necessary protein intake requirements, and establishing that the reference values (g of protein per kg of body weight per day) are as follows: Infants < 4 months: 2.5–1.4; children: 1.3–0.8; adults < 65 years: 0.8; adults > 65 years: 1.0 [13].

At the same time, to meet functional needs, such as promoting the accumulation of skeletal muscle proteins and physical strength, a dietary intake of 1.0, 1.3, and 1.6 g of protein per kilogram of body weight per day is recommended for individuals with minimal, moderate, and intense physical activity, respectively. Prolonged consumption of proteins at a rate of 2 g per kilogram of body weight per day is safe for healthy adults, and the tolerable upper limit is 3.5 g per kilogram of body weight per day for well-adapted subjects [14]. In this sense, it should also be considered that nutrient intake such as protein, carbohydrates, and lipids will vary according to the specific requirements of the sports discipline, as well as the exact timing of intake considering whether it is done before, during, or after exercise [15,16].

With regard to the consumption of branched-chain amino acid (BCAA), it has been recommended that athletes’ intake should be 200 mg/kg·day^−1^ to attenuate exercise-induced muscle damage [17]. However, there has been a call to pay particular attention from the healthcare team and consumers of these products because supplementation protocols vary widely in terms of timing and quantity. Additionally, BCAAs are also available in different supplementation products (e.g., whey protein) and are often combined with other nutrients (e.g., carbohydrates). Therefore, the potential benefits of supplementation with isolated BCAAs among athletes to attenuate muscle soreness and delay fatigue should be interpreted with caution [18].

In populations with the presence of comorbidities, it is highlighted that high chronic protein intake (>2 g per kilogram of body weight per day for adults) may lead to digestive, renal, and vascular abnormalities and should be avoided [14]. It has been studied that high protein consumption at an early age could lead to diseases such as obesity and non-communicable diseases; therefore, having a low-protein formula would allow for a reduction in body mass index (BMI) and the risk of childhood obesity, thus reducing the risk of developing obesity in adulthood.

Due to a lack of well-conducted dose–response trials in humans, national health agencies do not establish recommendations on the tolerable upper intake levels (UL) of amino acids. Recently, a publication suggested that, in relatively healthy adult individuals, tested amino acids are well-tolerated, and ULs, or the no-observed-adverse-effect level (NOAEL) and lowest-observed-adverse-effect level (LOAEL), can be determined [19]. Therefore, particular attention should be paid to the labeling appearing on the nutritional facts of products marketed in our country to provide safe intake according to the individual and sports requirements of each individual. 

Finally, the essential amino acid (EAA) requirements in different mammals are not identical, and ratios among them should be considered when projecting an efficient formulation. Additionally, genes respond to different qualities and quantities of nutritional supply. In this sense, non-essential amino acids (NEAA) can become ‘conditionally essential’ due to elevated needs during pathological conditions, and metabolism may not be able to maintain their concentrations at sufficient levels to match metabolic requirements. Therefore, maintaining an optimal balance is essential for ensuring optimal health and avoiding pathological conditions or manifestations of diseases [2].

To date, studies reporting the main unified nutritional contents contained in the products marketed in the country for sports purposes have not yet been carried out [20]. This article aims to identify and compare the information on the composition of micronutrients, macronutrients, and amino acid profiles reported in the protein shakes available in the Santiago de Chile market.

## 2. Materials and Methods

### 2.1. Ethics Statement

This study has been performed in keeping with the latest version of the Declaration of Helsinki, in accordance with Chilean legislation. The ethical committee approval was not required as no interventions were performed on humans. The study accessed sports nutritional supplements available in the Santiago de Chile market.

### 2.2. Study Design

This research was designed as a descriptive, cross-sectional, and observational study, and it was written in accordance with the guidelines of Observational Descriptive Studies as Research Designs (The MInCir Initiative) [21]. The study started in July 2021. All available websites offering online sales of nutritional products for sports purposes in Chile were consulted. The analysis focused on the period from 1 September 2021 to 31 December 2022.

### 2.3. Data Extraction

Each of the supplements available in the Chilean online market was individually identified. The information was accessed following the new nutrition facts label recommendations of the Food and Drug Administration [22]. Dietary supplement labels were required to include the name and location information of the manufacturer or distributor. If more information was required about the dietary supplement, the manufacturer or distributor had to be consulted directly for information that supported the product’s claims and the safety and efficacy of its ingredients. During the data collection process, there was a reviewer (CJ) who specifically extracted the nutritional information contained in the labels of sports nutritional supplements. A second (PP) and third reviewer (JF) then verified that the information captured was correct. If there were differences, a fourth reviewer (GD) would be in charge of reaching an agreement. Once the data from the review of nutritional labeling was obtained from the web pages of companies, distributors, or manufacturers, as well as face-to-face sales sites, we proceeded to directly consult the companies via email and telephone when researchers had doubts about the reported nutritional information.

Within the selection criteria, products with protein content were included without distinction of brands or prices, as long as they were available for sale on the Chilean online market. The presentation format included powder to dilute, both with and without the addition of amino acids. Furthermore, it should be noted that the sports nutritional supplements were sourced without discrimination based on origin. In other words, products from manufacturers located in the following geographical areas were sought: North and South America, Africa, Asia, Europe, and Oceania.

A categorization was carried out at three levels. The first were sports nutritional supplements that contained protein of animal origin as the main macronutrient. Among these, whey protein stood out, in its different forms and proportions, such as whey concentrate, isolated, and/or hydrolyzed whey, with or without the addition of other components such as BCAAs, calcium caseinate, micellar casein, and egg white albumin. In addition, there were sports nutritional supplements of animal origin with ingredients that come from beef protein and others that only contain egg white albumin protein. The second hybrid category was a mixture of animal and vegetable proteins. In this category were sports nutritional supplements containing whey plus rice, and/or soy protein. And the third category was of solely vegetable origin, with the content of its ingredients coming from protein from peas, rice, and hemp. Therefore, this category was suitable for consumption in a vegan eating style. Specifically, the total energy intake in kilocalories (kcals), the number of macronutrients in grams (proteins, carbohydrates, and lipids), and the number of micronutrients in milligrams, focusing only on the minerals potassium and sodium, were identified.

The energy content was segmented into 5 levels (0–350; 351–400; 401–450; 451–500; 500 and more kcal), and the concentration of protein in grams per product was also pooled into 5 levels (50–60; 61–70; 71–80; 81–90; over 91 protein grams). The amino acid profile, organized according to groups, was recorded in a complete aminogram. Valine, Isoleucine, Leucine, L-Isoleucine, L-Leucine, L-Lysine, L-Methionine, L-Phenylalanine, L-Threonine, L-Tryptophan, L-Valine, L-Arginine, L-Cysteine, L-Tyrosine, L-Histidine, L-Proline, L-Glutamine, L-Aspartic Acid, L-Serine, L-Glycine, and L-Alanine were identified. All values were measured in milligrams per hundred grams of product.

### 2.4. Statistical Analysis

A descriptive analysis was performed. A percentage distribution was made according to the geographical area of origin. Likewise, the source of nutritional origin was determined in percentage terms. The data were treated by normalizing the distribution of the selected products, considering a product concentration of 100 g. The normal distribution of the data was calculated using the Shapiro–Wilk test. Given the non-parametric nature of the data, medians and the major and minor ranges were calculated. Cluster techniques were employed to ascertain the distribution of concentration for both the energy content and the amount of protein in grams among the selected products. A box plot was created to visualize the distribution, in milligrams, of the amino acids.

To organize and visualize the information, a table was created in an Excel spreadsheet, version number: 2403 (Microsoft Corporation by Impressa Systems, Santa Rosa, CA, USA), where all the data were recorded. All graphs and descriptive analyses were performed using R (The R Foundation for Statistical Computing, v. 3.6.2) and R Studio, v. 4.1.0.

## 3. Results

### 3.1. Products and Origin

**Figure 1:**
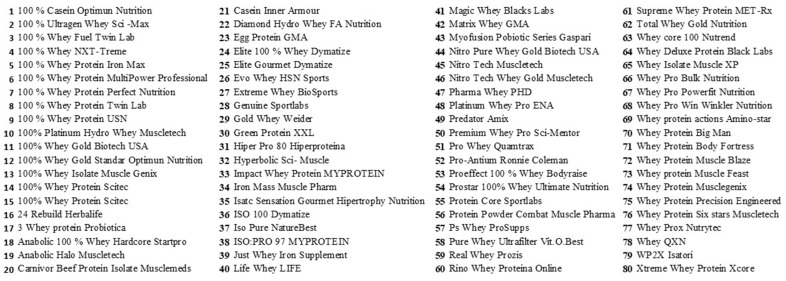
List of selected products.

Eighty protein shakes for human consumption from different brands and manufacturers sold on the Chilean online market were chosen for analysis. Figure 1 presents a comprehensive list of the selected products, arranged in alphabetical order.

**Figure 2:**
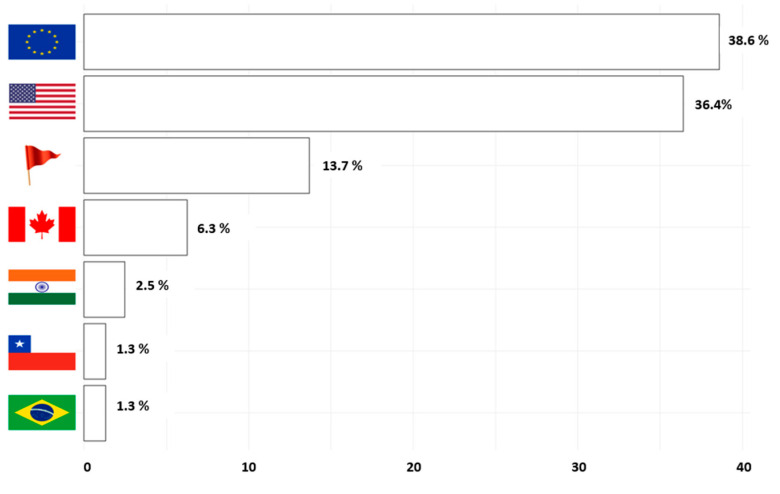
Distribution of the origin of the available products. From top to bottom, the markets are Europe, the United States, no information, Canada, India, Chile, and Brazil.

It was observed that a significant majority (over 80%) of the products available on Chilean online market originate from the European and North American markets, while the participation of the Asian and South American markets is relatively minimal. Notably, approximately 15% of the products have undisclosed origin with no reported information. Figure 2 provides information about the origin of the selected products.

### 3.2. Presentation

Selected product presentations feature a diverse range of designs, sizes, shapes, and colors, all aimed at capturing the customer’s attention. The majority of these products are packaged in plastic containers with lids, as this is the industry standard. Additionally, they are also available in bag format and smaller quantities within compact boxes. However, there is one brand that stands out by offering individual sachets per portion. It is important to note that all the brands ensure the hermetic sealing of their packaging to preserve the products’ organoleptic properties. Also, all the products were measured in scoops, with varying gram dosages based on the available products on Chilean online market. The median dosage among the protein shakes was 32 g (range from 25 to 52). Moreover, it is worth highlighting that the majority of products had a dosage of 30 g per scoop.

### 3.3. Composition

**Figure 3:**
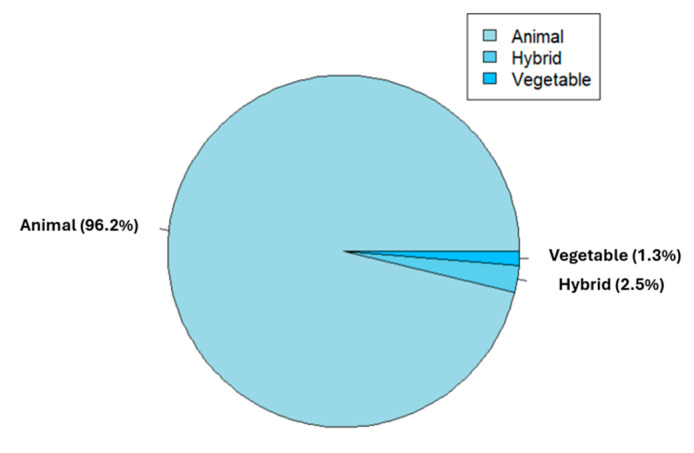
Protein composition of the available products.

Overall, the analysis reveals that 96.3% of the protein shakes available on Chilean online market consist of animal-based protein as the primary macronutrient. Notably, within this category, 96.1% of the shakes contain whey protein in varying forms and proportions. A smaller proportion, 2.6%, is derived from beef protein, while only 1.3% exclusively contains egg white albumin protein. Additionally, the protein shakes stand out for their inclusion of concentrated whey, isolated and/or hydrolyzed whey, and the presence of other components such as BCAAs, calcium caseinate, micellar casein, and egg white albumin. In contrast, mixed supplements, which combine animal and vegetable-based proteins (such as rice and soy), account for 2.5% of the protein shakes available on the Chilean online market. Finally, a mere 1.3% of the protein shakes are suitable for consumption within a vegan dietary style, as they are composed of plant-based proteins derived from peas, rice, and hemp. Figure 3 shows the composition of the supplements available on the Chilean online market.

### 3.4. Macronutrients and Minerals

After normalizing the dosage values to 100 g per product, the energy kcal distribution based on the quantity of protein supplements was predominantly clustered around 400 kcal. Specifically, the combined macronutrients (proteins, carbohydrates, and fats) provided a median energy value of 390 (range from 312 to 514) kcal.

**Figure 4:**
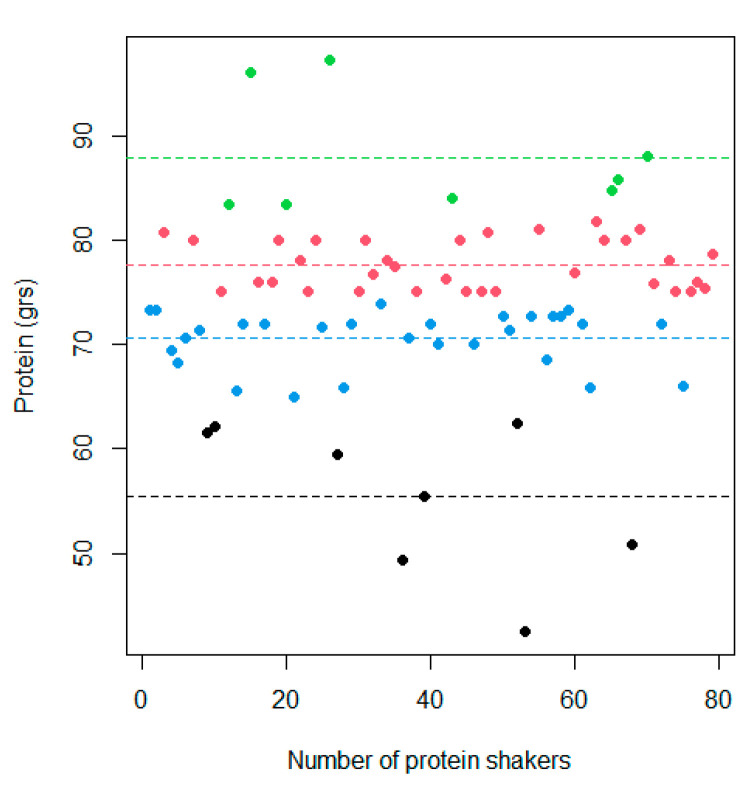
Protein distribution by protein shakes.

Additionally, it was observed that the distribution of protein concentration, measured in grams per 100 g of product, for the available protein supplements on the Chilean online market, was predominantly concentrated in the cluster segment of 70 to 80 g, as depicted in Figure 4. The median protein content per 100 g of product was found to be 75 (range from 42.5 to 97.2) g.

**Figure 5:**
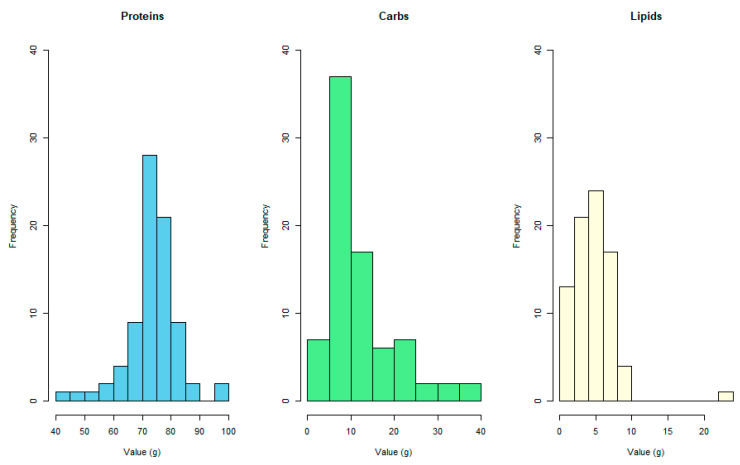
Macronutrient distribution by protein shakes.

Furthermore, the concentration of carbohydrates in the selected product sample had a median value of 9.63 (range from 0 to 38.2) g of this macronutrient per 100 g of product. Similarly, the determined lipid concentration showed a median of 4.55 (range from 0 to 22.3) g. Figure 5 provides a comparative view of the macronutrients presented in the selected protein supplements, highlighting their respective protein, carbohydrate, and lipid content.

Only two minerals were identified in the sample of analyzed supplements. The sodium concentration amounted to a median of 246.21 (range from 0 to 2400) mg per 100 g of supplement. On the other hand, the concentration of potassium in the same measurement showed a median of 0 (range from 0 to 1212) mg.

### 3.5. Amino Acids

**Figure 6:**
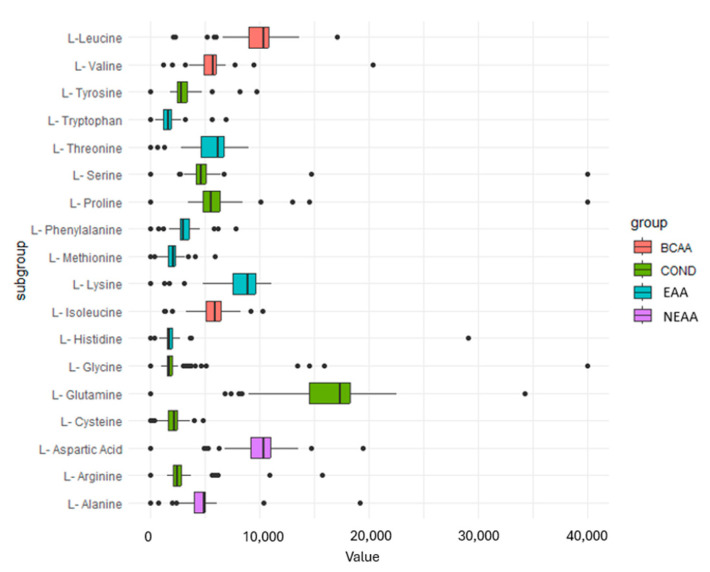
Distribution of amino acid concentration by group. BCAA, Branched-Chain Amino Acids; COND, conditional amino acid; EAA, essential amino acid; NEAA, no essential amino acid.

Finally, a total of 18 amino acids were identified in the selected protein supplements. Among them, 16.7% corresponded to BCAAs, 33.3% were essential amino acids, 38.9% were classified as conditional amino acids, and 11.1% were non-essential amino acids. The median concentration of all amino acids combined was 4749.75 mg. The lowest concentration was found in the essential amino acid L-Tryptophan with 1591.50 mg, while the conditional amino acid L-Glutamine had the highest median concentration at 17,336 mg. When examining the distribution by group, it was found that, among the BCAAs, L-Leucine exhibited the highest concentration with 10,386 mg (range from 2030 to 17,083 mg). Within the conditional amino acids, as previously described, glutamine showed the highest concentration. Among the essential amino acids, L-Lysine displayed a higher concentration with 8897 mg (range from 0 to 1110 mg), and, finally, for the non-essential amino acids, Aspartic Acid had the highest concentration with 10,359 mg (range from 0 to 19,450 mg). Figure 6 displays the distribution of median concentrations for each amino acid based on their respective groups.

## 4. Discussion

The main objective of this study was to describe and compare the macronutrient content and AA profile of eighty high-protein sports nutritional supplements available on the Chilean online market. Specific quantities of each substance reported for each of the analyzed supplements were determined, allowing for comprehensive information to be provided to individuals or professionals who wish to consume or prescribe these products considering the energy, sports, nutritional, and/or biological demands of individuals. This study offers a novel referencing proposal to generally consider the concentration levels of nutritional composition presented by these supplements in the national market.

Both endurance athletes and power athletes widely accept that they should consume more protein than the recommended daily intake. However, considering the variety of proteins available, much less is known about the benefits of consuming one protein over another [23]. The findings of this study revealed that the analyzed supplements had a higher amount of conditional AA, followed by EAA, with the highest concentration specifically found in L-Glutamine and L-Leucine, both of which belong to the BCAA group of AA at the expense of NEAA. This preference for supplements high in EAA and conditional AA has health effects that people should think about. For example, an imbalance of EAA in the diet or antagonism between them could often cause toxicity. However, dietary intake recommendations still do not provide data on the maximum tolerable intake levels of EAA in the diet for infants, children, or adults [24]. For instance, reports indicate that species, race, age, physiological status, and disease state, in addition to the availability of EAA and glucose, influence the rates of NEAA synthesis [25]. 

Additionally, various reports support the involvement of NEAA in the prevention of many diseases and disorders, including cancer [26]. On the other hand, it has been documented that the excessive consumption of essential amino acids could have physiological consequences for the body. Specifically, changes in circulating levels of EAA, particularly an increase in BCAA, have been identified in obese human and animal models. However, reports on the effects and underlying mechanisms of dietary imbalances of EAA on human body weight are scarce, and further research is needed in the future [27]. Therefore, it should be considered that the availability of an AA for synthesis or consumption is the result of a complex interaction between tissue-specific gene expression programs, dietary intake, and local consumption and secretion rates [28]. 

Gaining a comprehensive understanding of the nutritional composition of protein supplements available in this market is particularly important. This information will prove invaluable for professionals and individuals interested in using these products for better sports planning and informed decision-making, tailored to the specific needs of athletes and the general population. For instance, a study by Burke in 2012 [29] highlighted the positive impact of consuming various protein sources on plasma amino acid profiles in athletes, both at rest and after exercise. Notably, liquid whey protein-based supplements demonstrated higher levels of total AA, EAA, BCAAs, and Leucine compared to other protein sources. Moreover, it is crucial to emphasize the significance of the postprandial responses of plasma AA concentrations, as they profoundly affect post-exercise muscle protein synthesis. The availability of EAA plays a vital role in this response. Therefore, solely supplementing with BCAA-containing amino acids might not be optimal, as sufficient substrate availability is essential to achieve optimal rates of post-exercise muscle protein synthesis [30]. However, particular attention must also be given to the doses and contents of the products, and, despite the limited information about doses or UL levels, some studies have determined certain indicators should be considered. These include UL levels for Leucine in young adults (35 g/day), Tryptophan (4.5 g/day), and Leucine in elderly individuals (30 g/day); NOAEL and LOAEL for Methionine at 3.2 and 6.4 g/day, respectively; NOAEL for Arginine (30 g/day); NOAEL and LOAEL for Lysine at 6 and 7.5 g/day, respectively; NOAEL and LOAEL for Histidine at 8 and 12 g/day, respectively; and NOAEL for Phenylalanine (12 g/day), Serine (12 g/day), Ornithine (12 g/day), and Citrulline (24 g/day) [19].

Another crucial factor to consider is the protein content of sports nutritional supplements combined with BCAAs, particularly those of animal origin containing whey protein. These supplements have been shown to significantly increase post-exercise muscle protein synthesis due to the presence of intact whey protein, which provides essential amino acids essential for synthesis. However, it is important to note that relying solely on Leucine supplementation can activate the metabolic pathway that oxidizes all BCAAs, resulting in reduced concentrations of Valine and Isoleucine. This, in turn, becomes a limiting factor for muscle protein synthesis [31]. As a result, selecting the appropriate nutritional product becomes a crucial element in the planning process, aligning with the sports objectives set by the athlete and their team.

This article is subject to certain limitations. Firstly, accessing detailed nutritional information and ingredient content of sports nutritional supplements poses challenges. Despite the wide variety of protein supplements available in Chilean online market, which can be found in pharmacies, online stores, and nutritional stores. This limitation restricts individuals from making well-informed and appropriate choices, tailored to their specific objectives, or as prescribed by sports nutrition professionals. Part of the difficulty may stem from the current national legislation, which does not mandate the transparent disclosure of information about the content and ingredients of these products for commercialization.

The article has a second limitation related to the collection of information on the macronutrients and AA profile of the protein supplements. The authors found that the information they could gather is solely based on what the manufacturers disclose through product labeling or direct contact with distributors. This limitation stems from Article 535 of Title XXIX, paragraph 1, of the Chilean Food Sanitary Regulations, which only requires the mention of dietary ingredients for food supplements. These dietary ingredients are substances intentionally used to supplement the human diet, such as vitamins, minerals, amino acids, lipids, dietary fiber, or other naturally present elements in food. They must comply with quality and safety specifications. However, the current regulations do not mandate protein shakes to undergo bromatological analysis to verify their content and ingredients before approval and entry into the Chilean online market. As a result, consumers must rely solely on the information provided by the manufacturers on the label, including details regarding the ingredients, nutritional information, protein content, and amino acid profile.

Another limitation of this study is that the protein shakes available on the Chilean online market do not fully comply with the provisions stated in Title XXIX, paragraph 2, article 40 of the Food Sanitary Regulations. According to this regulation, products must meet specific nutritional properties and display the label “Food for sports” with an appropriate descriptor, such as “Good source of protein”. This label should be prominently placed on the main panel of the container, with easily legible letters in a contrasting color to the label’s background. It indicates that the food provides between 20% and 39% of the recommended daily dose of proteins, ensuring optimum quality and digestibility. During the analysis, it became evident that the majority of the products available on the market are imported, and they might not fully adhere to the requirements outlined in the national regulatory framework. These products often rely primarily on the labeling laws of their country of origin, which might differ from Chilean regulations, yet they are still accepted for marketing in Chile.

Finally, the last and major limitation of this study was that the authors did not specifically assess the macro- and micronutrient content. Essentially, the main goal was to identify and compare the information on the composition of micronutrients, macronutrients, and amino acid profiles reported in the protein shakes available in the Santiago de Chile market. However, this study provides an initial approach to the current state of protein shake products marketed in our country, allowing for further studies to thoroughly evaluate the content reported by the industries that distribute them. Additionally, it provides valuable information for consumers, athletes, individuals with comorbidities, medical professionals, healthcare practitioners, and governmental entities to have more information for decision-making purposes.

## 5. Conclusions

Finally, an abundance of high-calorie animal products stands out, particularly protein supplements with elevated concentrations of the amino acids L-Glutamine and L-Leucine. It is essential to highlight that the increasing consumption of these products among both the global and Chilean populations has underscored the necessity for a reliable tool to aid in selecting appropriate nutritional prescriptions to enhance sports performance. In future studies, conducting bromatological analyses of the protein shakes to verify their contents can be beneficial. Subsequent analysis of the postprandial responses obtained with these nutritional supplements, both after exercise and at rest, can provide concrete results, confirming their efficacy in promoting an elevated rate of muscle protein synthesis in consumers.

## Data Availability

Data are contained within the article.
